# Functional characterization of rare FOXP2 variants in neurodevelopmental disorder

**DOI:** 10.1186/s11689-016-9177-2

**Published:** 2016-11-28

**Authors:** Sara B. Estruch, Sarah A. Graham, Swathi M. Chinnappa, Pelagia Deriziotis, Simon E. Fisher

**Affiliations:** 1 Language and Genetics Department, Max Planck Institute for Psycholinguistics, Wundtlaan 1, 6525 XD Nijmegen, the Netherlands; 2 Donders Institute for Brain, Cognition and Behaviour, Radboud University, 6525 EN Nijmegen, the Netherlands

**Keywords:** Transcription factor, Speech, Language, Functional genetics, Neuroscience

## Abstract

**Background:**

Heterozygous disruption of *FOXP2* causes a rare form of speech and language impairment. Screens of the *FOXP2* sequence in individuals with speech/language-related disorders have identified several rare protein-altering variants, but their phenotypic relevance is often unclear. *FOXP2* encodes a transcription factor with a forkhead box DNA-binding domain, but little is known about the functions of protein regions outside this domain.

**Methods:**

We performed detailed functional analyses of seven rare FOXP2 variants found in affected cases, including three which have not been previously characterized, testing intracellular localization, transcriptional regulation, dimerization, and interaction with other proteins. To shed further light on molecular functions of FOXP2, we characterized the interaction between this transcription factor and co-repressor proteins of the C-terminal binding protein (CTBP) family. Finally, we analysed the functional significance of the polyglutamine tracts in FOXP2, since tract length variations have been reported in cases of neurodevelopmental disorder.

**Results:**

We confirmed etiological roles of multiple FOXP2 variants. Of three variants that have been suggested to cause speech/language disorder, but never before been characterized, only one showed functional effects. For the other two, we found no effects on protein function in any assays, suggesting that they are incidental to the phenotype. We identified a CTBP-binding region within the N-terminal portion of FOXP2. This region includes two amino acid substitutions that occurred on the human lineage following the split from chimpanzees. However, we did not observe any effects of these amino acid changes on CTBP binding or other core aspects of FOXP2 function. Finally, we found that FOXP2 variants with reduced polyglutamine tracts did not exhibit altered behaviour in cellular assays, indicating that such tracts are non-essential for core aspects of FOXP2 function, and that tract variation is unlikely to be a highly penetrant cause of speech/language disorder.

**Conclusions:**

Our findings highlight the importance of functional characterization of novel rare variants in FOXP2 in assessing the contribution of such variants to speech/language disorder and provide further insights into the molecular function of the FOXP2 protein.

**Electronic supplementary material:**

The online version of this article (doi:10.1186/s11689-016-9177-2) contains supplementary material, which is available to authorized users.

## Background

FOXP2 is a member of the forkhead box (FOX) family of transcription factors and has crucial roles in the development of the brain and other organs [1, 2]. Heterozygous disruptions of the *FOXP2* gene cause a rare and severe speech and language disorder (OMIM 602081) [3]. This disorder was first reported in a three-generation pedigree (the KE family), in which approximately half of the individuals have difficulties with learning to make the co-ordinated orofacial movements required for speech (childhood apraxia of speech, CAS), together with wide-ranging impairments in comprehension and production of spoken and written language, but without major deficits in other aspects of cognitive functioning [4]. All affected members of the family were found to carry a missense variant in *FOXP2* that alters a critical residue within the DNA-recognition helix of the FOX domain and thus prevents DNA binding and regulation of transcription [4–6]. A number of individuals have since been reported to present with severe speech/language difficulties together with heterozygous whole gene deletions or chromosomal translocations disrupting *FOXP2*, confirming the necessity of two functional copies of this gene for typical speech and language development [3].

Screening of the *FOXP2* coding region for protein-altering variants has been performed in a few small cohorts of children with speech articulation disorders similar to those reported in the KE family [7–9]. In addition, to address the possibility that *FOXP2* disruption might also be a factor in other disorders characterized by speech/language problems, similar screens have been performed in individuals with specific language impairment, speech sound disorder, autism, schizophrenia, and epilepsy of the speech cortex [10–18]. There are no common non-synonymous variants in FOXP2 in the general population, and relatively little coding sequence variation has been observed in individuals with speech/language-related disorders, indicating that *FOXP2* disruptions are a rare cause of such disorders, which likely have a highly heterogeneous genetic basis. Nonetheless, screening for *FOXP2* variants in individuals with neurodevelopmental phenotypes has identified a small number of rare protein-altering variants, including five missense variants, one stop-gain variant, one 2-bp deletion resulting in a frameshift, and several in-frame insertions or deletions of glutamine residues within polyglutamine tracts. However, the contribution of individual rare variants to disorder often remains unclear because the genetic evidence in isolation is insufficient to confirm a causal or contributory role, and the effect of the variant on protein function is unknown.

To clarify the etiological contribution of the rare FOXP2 variants reported to date in individuals with neurodevelopmental disorders, we performed functional characterization of these variants by assaying their effects on a range of molecular properties. In addition, we characterize the interaction between FOXP2 and the co-repressors of the C-terminal binding protein (CTBP) family, which may have a central role in FOXP2-mediated transcriptional repression. Finally, we provide the first detailed examination of the role of the polyglutamine tract in FOXP2 function, in order to shed light on the contribution of tract length variation to disorder.

## Methods

### DNA constructs

The cloning of human FOXP2 (NM_014491), FOXP1 (NM_032682), and CTBP1 (NM_001328) and mouse Foxp2 (NM_053242) has been described previously [6, 19]. The coding sequence of CTBP2 (NM_001329) was amplified from human foetal brain cDNA using the primers listed in Additional file 1. The FOXP2 p.Q17L, p.M406T, p.P416T, p.R553H, p.N597H, p.N303T, p.S325N, and p.N303T/p.S325N variants were generated by site-directed mutagenesis using the Quick-Change Site-Directed Mutagenesis kit (Stratagene) following the manufacturer’s protocol. (Note that the numbering of FOXP2 variants throughout this manuscript is given with respect to the coding sequence of the predominant isoform, NM_014491, which normally encodes a 715 amino-acid protein.) Primers used in site-directed mutagenesis are listed in Additional file 2. The FOXP2 p.R328* variant and synthetic truncated forms of FOXP2 were generated using the primers listed in Additional file 1. Synthetic FOXP2 variants with reduced polyglutamine tracts were generated using a PCR-based strategy [20]. All FOXP2 variants were initially generated in an intermediary plasmid, pCR2.1-TOPO (Life Technologies), and the entire coding sequence of FOXP2 was verified by Sanger sequencing before being subcloned into the final expression vector. For expression of fusion proteins with *Renilla* luciferase, yellow fluorescent protein (YFP), mCherry, and three tandem N-terminal Myc tags, cDNAs were subcloned into the pLuc, pYFP, pmCherry, and pMyc expression vectors, respectively, which have been described previously [19–21]. The SRPX2 luciferase reporter plasmid was generated by subcloning a 1146-bp region of the SRPX2 promoter into the promoterless firefly luciferase vector pGL4.23 (Promega), as described previously [21]. All constructs were verified by Sanger sequencing. Plasmid sequences are available upon request.

### Cell culture and transfection

HEK293 were obtained from ECACC (cat. no. 85120602) and cultured in DMEM supplemented with 10% foetal bovine serum. Transfections were performed using GeneJuice (Merck-Millipore) according to the manufacturer’s instructions.

### Western blotting

HEK293 cells were transfected in 6-well plates and cultured for 48 h. Cells were lysed for 10 min at 4 °C with 100 mM Tris pH 7.5, 150 mM NaCl, 10 mM EDTA, 0.2% Triton X-100, 1% PMSF, and protease inhibitor cocktail. Cell lysates were cleared by centrifugation at 10,000×*g* for 3 min at 4 °C. Proteins were resolved on 10% SDS-polyacrylamide gels and transferred to PVDF membranes using a TransBlot Turbo Blotting apparatus (Bio-Rad). Membranes were blocked in PBS containing 5% milk and 0.1% Tween-20 and incubated overnight at 4 °C with primary antibody. The following antibodies were used: anti-GFP (Clontech cat. no. 632380, 1:8000, for YFP constructs); anti-Myc tag (Abcam cat. no. ab9106, 1:1000); and anti-β-actin (Sigma cat. no. A5441, 1:10,000). After washing, membranes were incubated with horseradish peroxidase-conjugated goat anti-mouse or anti-rabbit IgG for 45 min at room temperature. Proteins were visualized using Novex ECL Chemiluminescent Substrate Reagent Kit (Invitrogen) and a ChemiDoc XRS+ imaging system (Bio-Rad).

### BRET assay

Bioluminescence resonance energy transfer (BRET) assays were performed as described [19]. Briefly, HEK293 cells were transfected in 96-well plates with DNA plasmids encoding YFP- and luciferase-fusion proteins. After 36–48 h, Enduren Live Cell Luciferase Substrate (Promega) was added at a final concentration of 60 μM. Cells were cultured for a further 4 h, and emission readings (integrated over 10 s) were taken using a TECAN F200PRO microplate reader using the Blue1 and Green1 filter sets. Expression levels of the YFP-fusion proteins were measured by taking fluorescent readings using the filter set and dichroic mirror suitable for green fluorescent protein (excitation 480 nm, emission 535 nm). The corrected BRET ratio was calculated with the following formula: [Green1_(experimental condition)_/Blue1_(experimental condition)_] − [Green1_(control condition)_/Blue1_(control condition)_]. The control conditions used luciferase or YFP fused to a C-terminal nuclear localization signal.

### Fluorescence microscopy

HEK293 cells were seeded on coverslips coated with poly-l-lysine. Cells were cultured for 30 h post-transfection and then fixed with methanol. Nuclei were stained with Hoechst 33342. Fluorescence images were acquired using an Axiovert A-1 fluorescent microscope with ZEN Image software (Zeiss).

### Fluorescence-based quantitation of protein expression levels

HEK293 cells were transfected in triplicate with YFP-tagged FOXP2 variants and mCherry, in clear-bottomed black 96-well plates. Cells were cultured at 37 °C with 5% CO_2_ in a TECAN M200PRO microplate reader equipped with a Gas Control Module for live-cell kinetic assays. Fluorescence intensity was measured 48 h post-transfection. For each well and time point, the background-subtracted YFP intensity was divided by the background-subtracted mCherry intensity. Triplicate conditions were averaged.

### Luciferase reporter assays

HEK293 cells were seeded in clear-bottomed white 96-well plates and transfected in triplicate. For the SV40 assay, cells were transfected with 12 ng of pGL3-promoter firefly luciferase reporter construct containing the SV40 promoter (Promega), 5 ng of pRL-TK *Renilla* luciferase normalization control (Promega), and 16 ng of YFP-FOXP2 (wild-type or variant) or YFP control construct. For the SRPX2 assay, cells were transfected with 4.3 ng of SRPX2 luciferase reporter construct, 5 ng of pGL4.74 *Renilla* luciferase normalization control (Promega), and 45 ng of YFP-FOXP2 (wild-type or variant) or YFP control construct. After 48 h, luciferase activity was measured in a TECAN F200PRO microplate reader using the Dual-Luciferase Reporter Assay system (Promega).

### Statistical analysis

The statistical significance of the luciferase reporter assays and BRET assays was analysed using a one-way analysis of variance (ANOVA) followed by Bonferroni’s post hoc correction.

## Results

### Rare FOXP2 variants implicated in neurodevelopmental disorder

**Fig. 1:**
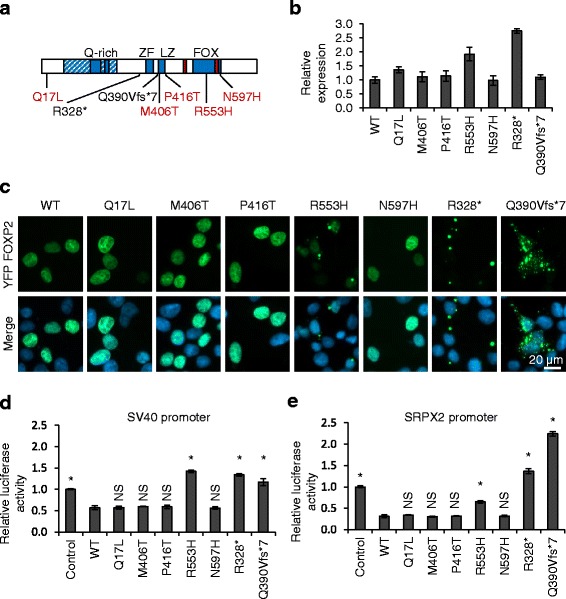
Functional characterization of rare FOXP2 variants. **a** Schematic representation of the FOXP2 protein showing rare variants found in individuals with neurodevelopmental disorders. Stop-gain and frameshift variants are shown in black and missense variants in *red*. Known domains are labelled: glutamine-rich (*Q-rich*) region (hatched shading) including polyglutamine tracts (*solid shading*), zinc finger (*ZF*), leucine zipper (*LZ*) and forkhead domain (*FOX*). Nuclear localization signals are indicated with *red bars*. **b** Fluorescence-based measurement of FOXP2 expression level. HEK293 cells were transfected with YFP-FOXP2, together with mCherry for normalization. Fluorescence intensity was measured 48 h post-transfection. Values are mean YFP/mCherry fluorescence ratios ± S.D. (*n* = 3), expressed relative to the value for wild-type (WT) FOXP2. **c** Fluorescence micrographs of HEK293 cells transfected with YFP-FOXP2. Nuclei were stained with Hoechst 33342. **d**, **e** Luciferase reporter assays for transcriptional regulatory activity of FOXP2. HEK293 cells were transfected with a firefly luciferase reporter vector containing the SV40 promoter (**d**) or the human *SRPX2* promoter (**e**), together with a *Renilla* luciferase normalization plasmid and YFP-FOXP2, or YFP alone (control). Values are mean relative luciferase activity ± S.D. (*n* = 3), expressed relative to the control. *Asterisks* indicate significant differences compared to wild-type (WT) FOXP2 (*p* < 0.05, one-way ANOVA followed by Bonferroni post hoc correction). *NS* not significant. Exact *p* values for both the SV40 and the *SRPX2* assays are <0.0001 for the control and the R553H, R328*, and Q390Vfs*7 variants, and >0.9999 for all other variants

We examined seven rare FOXP2 variants that have been observed in individuals with neurodevelopmental disorders, including five missense variants, one stop-gain variant, and one frameshift variant (Table 1, Fig. 1a). For three of the variants examined (p.N597H, p.P416T, p.Q390Vfs*7), this is the first report of any functional characterization. The remaining four variants have been studied previously to varying extents and are included here for comparison with the uncharacterized variants. All of the variants were additionally characterized using novel assays that have not been used to study FOXP2 in prior literature. Two of the variants are of particular interest because of their uncertain significance with regard to the phenotype of the affected cases. The p.N597H variant was found by targeted sequencing in a proband with CAS and was described as a likely pathogenic variant, but it was not ascertained if this variant occurred de novo, and no functional characterization was performed [7]. The p.M406T variant was identified in a proband with an epilepsy-aphasia spectrum disorder (focal epilepsy with continuous spike-and-waves during sleep), cognitive and language deficits, and polymicrogyria of the left rolandic operculum [15]. The variant was also carried by two siblings who were not known to have any neurological abnormality and was inherited from the father, who did not display any neurological or MRI abnormalities. It was suggested that this variant plays an etiological role in the epilepsy-aphasia spectrum disorder observed in the proband, acting as a risk factor with incomplete penetrance [15]. However, the contribution of this variant to the phenotype is tentative because of the lack of segregation with the disorder, the atypical phenotypic presentation in comparison to other cases of *FOXP2* disruption, and the limited functional characterization performed to date.

**Table 1:** Rare FOXP2 variants in individuals with neurodevelopmental disorders

Variant description^a^	Frequency in ExAC^b^	Phenotype and inheritance pattern	Role in disorder^c^	Reference
p.Q17L (missense) c.50A > T chr7:114426561A > T rs201649896	56/120570 (1 homozygote)	Found in a proband with CAS but not in an affected sibling. Parental genotypes not determined	Probably incidental	MacDermot et al. [8]
p.M406T (missense) c.1217 T > C chr7:114653960 T > C no rs ID	Not observed	Found in a proband with rolandic epilepsy and polymicrogyria, in two unaffected siblings and in unaffected father	Uncertain significance	Roll et al. [15]
p.P416T (missense) c.1246C > A chr7:114653989C > A rs369313543	1/121328	Present in two siblings with severe stuttering. Absent in affected father. Inherited from mother, who does not stutter but has oral motor impairments	Probably incidental	Turner et al. [9]
p.R553H (missense) c.1658G > A chr7:114662075G > A rs121908377	Not observed	Segregates with CAS in three generations of the KE family	Causal	Lai et al. [4]
p.N597H (missense) c.1789A > C chr7:114663469A > C no rs ID	1/120986	Found in a proband with CAS. Parental genotypes not determined	Uncertain significance	Laffin et al. [7]
p.R328* (stop-gain) c.982C > T chr7:114642616C > T rs121908378	Not observed	Present in a proband with CAS and in affected sibling. Inherited from affected mother	Causal	MacDermot et al. [8]
p.Q390Vfs*7 (frameshift) c.1168_1169del chr7:114652276_ 114652277del no rs ID	Not observed	De novo variant in a proband with sporadic CAS, dysarthria, and fine motor apraxia	Causal	Turner et al. [9]

^a^Variants are described in accordance with Human Genome Variation Society recommendations (www.hgvs.org/mutnomen, accessed June 2016) with reference to the major transcript NM_014491.3 (ENST00000350908). Genomic coordinates refer to the hg38 assembly. The rs ID number is provided for variants that are present in dbSNP

^b^Variant allele frequency in the Exome Aggregation Consortium (ExAC) dataset (http://exac.broadinstitute.org, accessed June 2016)

^c^Variants are described as causal if they segregate perfectly with childhood apraxia of speech (CAS) affection status, or if they occurred de novo in sporadic cases, and if they additionally have been demonstrated to cause loss of protein function, or are very likely to do so because of protein truncation. Variants are described as probably incidental if they do not segregate with CAS and are observed in the Exome Aggregation Consortium (ExAC) dataset. Other variants are described as of uncertain significance

Note: This table does not include newly described variants that were reported by Reuter et al. [58] after the completion of the present study

The seven FOXP2 variants were expressed as fusion polypeptides with YFP in HEK293 cells. The expression of these YFP-tagged variants (and of all other YFP-tagged forms of FOXP2 used in this study) was verified by western blotting, as shown in Additional file 3. Direct measurement of fluorescence intensity in live cells indicated that the missense variants and the truncated p.Q390Vfs*7 variant are expressed at broadly similar levels to the wild-type protein, whereas the p.R553H and p.R328* variants may have slightly increased expression (Fig. 1b). In cells of people carrying the p.R328* and p.Q390Vfs*7 variants, the expression levels of these protein variants are likely to be very low due to nonsense-mediated decay of the aberrant transcripts, although this has not been formally tested due to the lack of *FOXP2* expression in accessible tissue [8, 9]. The inclusion of these truncated variants in functional assays provides a useful comparison with missense variants because the truncated variants lack the DNA-binding domain and hence cannot function normally in transcriptional regulation.

### Effects of variants on nuclear localization and transcriptional regulation

The effect of the variants on FOXP2 localization was examined by direct imaging of the YFP-fusion proteins in transfected cells (Fig. 1c). Wild-type FOXP2 exhibited nuclear localization, with exclusion from nucleoli, as reported previously (Fig. 1c) [6, 22]. The two previously characterized etiological variants, p.R553H and p.R328*, showed disrupted nuclear localization and formation of aggregates, also consistent with earlier reports (Fig. 1c) [6, 22]. The uncharacterized Q390Vfs*7 variant formed cytoplasmic aggregates similar to those observed for the p.R328* variant, consistent with the loss of endogenous nuclear localization signals in these truncated variants (Fig. 1a,c) [22]. The p.Q390Vfs*7 variant therefore has a similarly deleterious effect on protein localization as the two known etiological variants. None of the other missense variants showed any sign of abnormal localization, including the putatively pathogenic p.N597H and p.M406T variants (Fig. 1c). Altered localization has been reported previously for the p.M406T variant, but in multiple independent experiments, we did not observe any loss of nuclear localization for this variant [15]. Thus, with the exception of the p.R553H variant, all the missense variants retain the nuclear localization necessary for transcriptional regulatory activity.

To assess the ability of the FOXP2 variants to regulate transcription, we performed luciferase reporter assays using the SV40 promoter, a viral promoter which is repressed by FOXP2 [6, 23]. The etiological p.R553H and p.R328* variants exhibited the expected loss of repression activity in this assay [6] (Fig. 1d). The uncharacterized p.Q390Vfs*7 variant showed a comparable loss of repression to the other etiological variants (Fig. 1f). In contrast, the remaining four missense variants showed similar activity to the wild-type protein (Fig. 1d). To verify these results using an endogenous human FOXP2 target gene, we performed luciferase reporter assays using a region of the *SRPX2* promoter [15]. As observed for the SV40 promoter, the truncated variants and the p.R553H variant showed loss of repressive activity, whereas the other missense variants did not differ significantly in activity from the wild-type protein (Fig. 1e). The p.M406T variant has previously been reported to show reduced transcriptional repression activity in relation to the *SRPX2* promoter [15]. Small differences between the promoter regions and vector backbones used in this and the previous study might account for these conflicting results. Nonetheless, our data indicate that the p.M406T variant does not exhibit a generalized reduction in transcriptional regulatory activity.

### Effects of variants on protein dimerization

FOXP2 and other proteins of the FOXP subfamily form dimers via their leucine zipper domains, a property which appears to be essential for transcriptional regulatory activity [24] (Fig. 1a). In FOXP3, loss of dimerization capacity as a result of an in-frame single amino acid deletion in the leucine zipper domain results in an immunological disorder known as IPEX syndrome (MIM 304790), with disease severity comparable to that resulting from loss of DNA-binding activity in FOXP3 [25, 26]. Variants affecting the dimerization of FOXP2 might therefore be a cause of speech/language disorder. Additionally, FOXP2 variants that have lost DNA-binding capacity but retain dimerization ability might interfere with the function of wild-type protein in cells of affected individuals, and thus exert a dominant-negative effect. Notably, FOXP2 not only forms homodimers but can also heterodimerize with FOXP1 and FOXP4, proteins which have partially overlapping expression patterns in the developing brain [24, 27–29]. (Note that FOXP3 is expressed only in haematopoietic cells.) Heterodimerization among certain FOXP proteins may therefore be an important mechanism for differential regulation of target genes in different neuronal subtypes. Of note, heterozygous disruption of *FOXP1* results in a severe neurodevelopmental phenotype that includes language deficits, potentially reflecting dysregulation of some of the same target genes impacted by *FOXP2* disruption [30, 31].

**Fig. 2:**
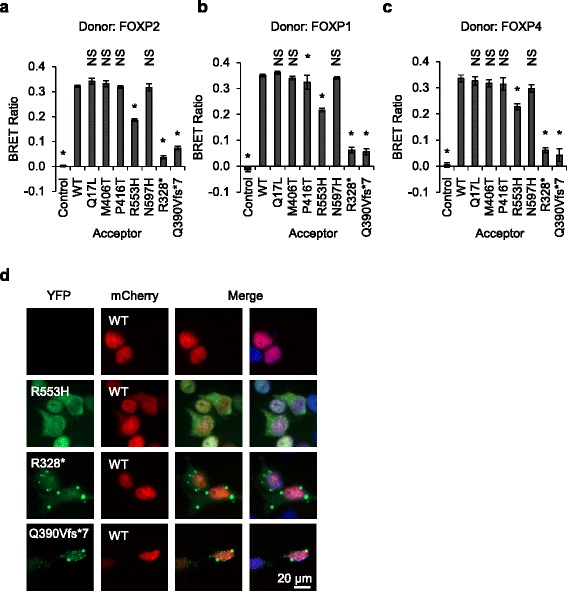
Interaction of rare FOXP2 variants with wild-type FOXP proteins. **a**–**c** Bioluminescence resonance energy transfer (*BRET*) assays for interaction of FOXP2 variants with wild-type (*WT*) FOXP proteins. HEK293 cells were transfected with YFP-FOXP2 variants (acceptor) and *Renilla* luciferase (donor) fusions of FOXP2 (**a**), FOXP1 (**b**) or FOXP4 (**c**). The control acceptor protein is a nuclear-targeted YFP. Values are mean corrected BRET ratios ± S.D. (*n* = 3). *Asterisks* indicate significant differences compared to wild-type (*WT*) FOXP2 (*p* < 0.05, one-way ANOVA followed by Bonferroni post hoc correction). *NS* not significant. Exact *p* values for **a** are <0.0001 for the control and the R553H, R328* and Q390Vfs*7 variants and >0.9999 for all other variants. Exact *p* values for **b** are <0.0001 for the control and the R553H, R328* and Q390Vfs*7 variants, 0.024 for the P416T variant, and >0.9999 for all other variants. Exact *p* values for **c** are <0.0001 for the control and the R553H, R328* and Q390Vfs*7 variants, 0.088 for the N597H variant, and >0.9999 for all other variants. **d** Fluorescence micrographs of HEK293 cells transfected with YFP-fusions of the FOXP2 variants p.R553H, p.R328* and p.Q390Vfs*7, together with wild-type FOXP2 fused to mCherry. Nuclei were stained with Hoechst 33342

Two of the five FOXP2 missense variants reported in individuals with neurodevelopmental disorders lie within or near the leucine zipper domain, which spans residues p.V388-L409 (Fig. 1a). These variants (p.M406T and p.P416T) could therefore affect FOXP2 dimerization. The interaction between FOXP2 variants and wild-type FOXP2 was assayed using a BRET assay, which enables protein-protein interactions to be monitored in live cells [19]. The interaction of the FOXP2 variants with wild-type FOXP1 and FOXP4 was also examined. As expected, wild-type FOXP2 was able to homodimerize and also to heterodimerize with FOXP1 and FOXP4 (Fig. 2a–c) [24]. The truncated p.R328* and p.Q390fs*7 variants were not able to dimerize with the wild-type proteins, consistent with the loss of the leucine zipper domain in these variants (Fig. 2a–c). Thus, even if these abnormal proteins are present in cells of affected individuals despite the activation of nonsense-mediated decay mechanisms, they could not interfere with the function of wild-type proteins via dimerization. In contrast, the p.R553H variant found in the KE family has a normal leucine zipper domain and showed only a modest reduction in interaction with wild-type FOXP2, FOXP1, and FOXP4 (Fig. 2a–c). Furthermore, co-transfection of wild-type FOXP2 with the p.R553H variant led to some wild-type protein being mislocalized to the cytoplasm (Fig. 2d). Mislocalization of wild-type protein was not observed upon co-transfection with the non-interacting variants p.R328* and p.Q390Vfs*7 (Fig. 2d). The p.R553H variant might therefore interfere with the function of wild-type FOXP2 in cells of people carrying this variant, and this effect could contribute to the phenotype observed in the affected members of the KE family. All four of the other missense variants generally demonstrated normal dimerization capacity (Fig. 2a–c). The p.P416T variant showed a statistically significant difference in interaction with FOXP1 (Fig. 2b), but this difference was not consistently observed across independent experiments. Therefore, despite being located in or near the leucine zipper domain, the p.M406T and p.P416T variants do not appear to affect FOXP2 dimerization and are unlikely to exert a pathogenic effect via this mechanism.

### Effects of variants on interaction with the co-repressors CTBP1 and CTBP2

**Fig. 3:**
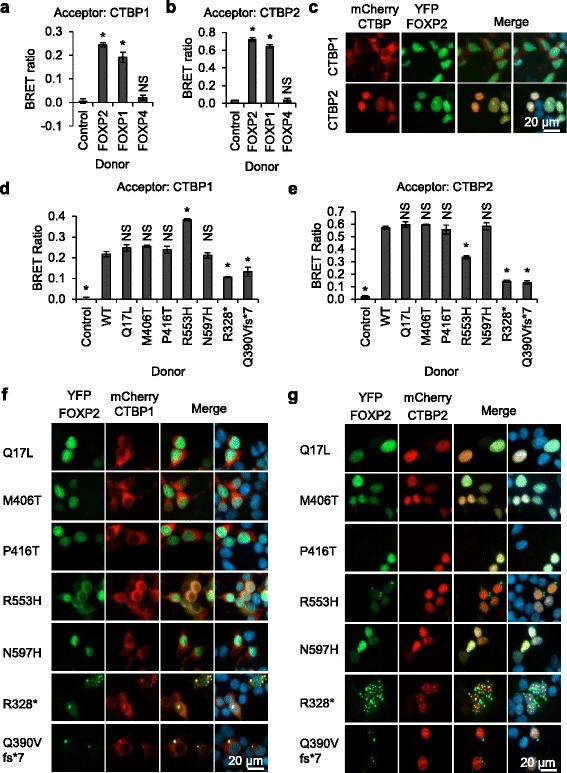
Interaction of rare FOXP2 variants with CTBP1/2. **a**, **b** BRET assays for interaction of FOXP proteins with CTBP1 and CTBP2. HEK293 cells were transfected with FOXP1, FOXP2, or FOXP4 fused to *Renilla* luciferase (donor) and CTBP1 (**a**) or CTBP2 (**b**) fused to YFP (acceptor). The control donor protein is a nuclear-targeted luciferase. Values are mean corrected BRET ratios ± S.D. (*n* = 3). *Asterisks* indicate significant differences compared to control (*p* < 0.05, one-way ANOVA followed by Bonferroni post hoc correction). *NS* not significant. Exact *p* values for both **a** and **b** are <0.0001 for FOXP2 and FOXP1 and >0.9999 for FOXP4. **c** Fluorescence micrographs of HEK293 cells transfected with mCherry-CTBP1 or mCherry-CTBP2, together with YFP-FOXP2. Nuclei were stained with Hoechst 33342. **d**, **e** BRET assays for interaction of rare FOXP2 variants with CTBP1 and CTBP2. HEK293 cells were transfected with FOXP2 variants fused to *Renilla* luciferase (donor) and CTBP1 (**d**) or CTBP2 (**e**) fused to YFP (acceptor). The control donor protein is a nuclear-targeted luciferase. Values are mean corrected BRET ratios ± S.D. (*n* = 3). *Asterisks* indicate significant differences compared to wild-type (*WT*) FOXP2 (*p* < 0.05, one-way ANOVA followed by Bonferroni post hoc correction). *NS* not significant. Exact *p* values for **d** are <0.0001 for the control and the R553H, R328* and Q390Vfs*7 variants, 0.221 for the M406T variant, and >0.9999 for all other variants. Exact *p* values for **e** are <0.0001 for the control and the R553H, R328* and Q390Vfs*7 variants and >0.9999 for all other variants. **f**, **g** Fluorescence micrographs of HEK293 cells transfected with YFP-FOXP2 and mCherry-CTBP1 (**f**) or mCherry-CTBP2 (**g**). Nuclei were stained with Hoechst 33342

A further mechanism by which variants in FOXP2 might lead to speech/language disorder is through disruption of protein-protein interactions between FOXP2 and crucial mediators of transcriptional regulation. Relatively, little is known about the interaction partners of FOXP2; however, proteins of the CTBP family have been identified as candidate FOXP2 interactors in multiple independent yeast two-hybrid screens [24, 32–34]. The CTBP family consists of two proteins, CTBP1 and CTBP2, which function as co-repressors for multiple transcription factors [35, 36]. The interaction between FOXP2 and CTBP1 has previously been validated by us using the BRET assay, and by others via co-immunoprecipitation, but the interaction with CTBP2 has not yet been confirmed [19, 24]. Using the BRET assay, we validated the interaction between CTBP2 and FOXP2 and found that this interaction produces a notably higher BRET signal than the interaction between CTBP1 and FOXP2 (Fig. 3a,b). The difference in the magnitude of the BRET signal may reflect subcellular localization differences between the CTBPs: CTBP2 is wholly nuclear and therefore shows a large degree of co-localization with wild-type FOXP2, whereas CTBP1 is found in both the cytoplasm and nucleus, and therefore has an overall lower degree of co-localization with FOXP2 (Fig. 3c) [37, 38]. Strikingly, we found that CTBP1 and CTBP2 are also able to interact with FOXP1, but not with FOXP4, in line with previously reported findings for CTBP1 (Fig. 3a,b) [24]. The lack of interaction between FOXP4 and CTBPs suggests that the FOXP proteins have partially divergent mechanisms for transcriptional regulation, which is particularly interesting given the ability of FOXP2 to dimerize with FOXP4 (Fig. 2c).

We next evaluated the effects of rare variants in FOXP2 on the interaction with CTBP1/2. The p.R553H variant found in the KE family showed slightly reduced interaction with CTBP2, but increased interaction with CTBP1, when compared with wild-type FOXP2 (Fig. 3d,e). Interestingly, the p.R553H variant displayed a mixed nuclear and cytoplasmic localization when transfected alone or with CTBP2, but became predominantly cytoplasmic in the presence of CTBP1 (Figs. 1e and 3f,g). In contrast, the nuclear localization of wild-type FOXP2 was not affected by co-transfection with CTBP1 (Fig. 3c). The cytoplasmic retention of the p.R553H variant, but not of wild-type FOXP2, upon co-transfection with CTBP1 may be a consequence of the loss of DNA-binding capacity in the p.R553H variant. These co-transfection experiments indicate that the p.R553H variant shows greater co-localization with CTBP1 but reduced co-localization with CTBP2, compared with wild-type FOXP2, consistent with the BRET data on interactions between these proteins (Fig. 3d,e). Although altered interaction with CTBP1/2 probably does not play a substantive role in the speech/language pathology in the KE family, the retained interaction between the p.R553H variant and CTBP1/2 is notable because it indicates that DNA-binding activity is not a prerequisite for interaction of FOXP2 with co-repressors of the CTBP family. The four other missense variants did not differ substantially from the wild-type protein in their interaction with CTBP1/2 in the BRET assay (Fig. 3d,e). These four variants thus have comparable properties to wild-type FOXP2 in assays of subcellular localization, transcriptional repression, protein dimerization, and interaction with co-repressor proteins, suggesting that all of these variants are benign rare polymorphisms (Table 2). In particular, we do not find any support for the etiological roles suggested previously for the p.M406T and p.N597H variants [7, 15].

**Table 2:** Summary of functional characterization of rare FOXP2 variants in individuals with neurodevelopmental disorders

Variant^a^	Localization	Transcriptional repression		Dimerization with FOXPs			Interaction with CTBPs		Role in disorder^b^
		SV40	SRPX2	FOXP2	FOXP1	FOXP4	CTBP1	CTBP2	
p.Q17L	+	+	+	+	+	+	+	+	Incidental
p.M406T	+	+	+	+	+	+	+	+	Incidental
p.P416T	+	+	+	+	+	+	+	+	Incidental
p.R553H	−	−	−	−	−	−	−	−	Causal
p.N597H	+	+	+	+	+	+	+	+	Incidental
p.R328*	−	−	−	−	−	−	−	−	Causal
P.Q390Vfs*7	−	−	−	−	−	−	−	−	Causal

+ Behaviour of the variant is comparable to that of the wild-type protein in this assay, − Behaviour of the variant differs from that of the wild-type protein in this assay

^a^Variants are described in accordance with Human Genome Variation Society recommendations (www.hgvs.org/mutnomen, accessed June 2016) with reference to the major transcript NM_014491.3 (ENST00000350908)

^b^Probable role of the variant in the disorder in the affected individual, based on the results of functional characterization

Interestingly, the p.R328* and p.Q390Vfs*7 variants partially retained the ability to interact with CTBP1/2, despite lacking a large proportion of the normal FOXP2 polypeptide (Fig. 3d,e). The interaction with CTBP1 in particular was only mildly affected (Fig. 3d). Furthermore, co-transfection of CTBP1 with the p.R328* and p.Q390Vfs*7 variants resulted in clear co-localization of CTBP1 with the truncated variants within cytoplasmic aggregates (Fig. 3d,f). The interaction with CTBP2 was more severely affected, but co-localization of CTBP2 and the p.R328* variant within aggregates was still readily apparent (Fig. 3e,g). The interaction between these severely truncated FOXP2 variants and CTBP1/2 suggests that some key determinants of CTBP binding lie near the N-terminus of FOXP2.

### Characterization of the interaction between FOXP proteins and CTBPs

**Fig. 4:**
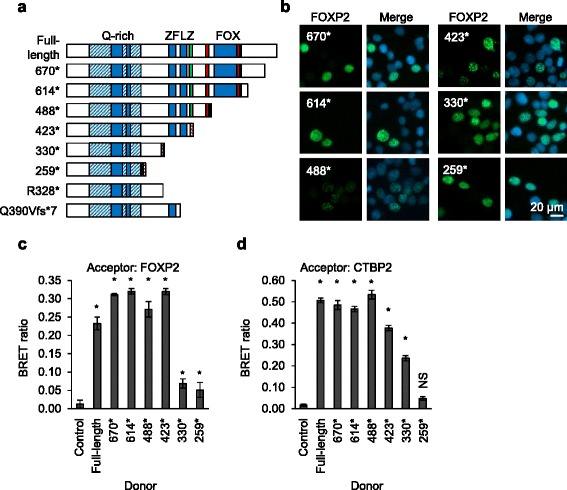
Mapping of the CTBP binding site in FOXP2. **a** Schematic representation of synthetic truncated forms of FOXP2. The R328* and Q390Vfs*7 variants identified in patients are included for comparison. Known domains are labelled: glutamine-rich (*Q-rich*) region (*hatched shading*) including polyglutamine tracts (*solid shading*), zinc finger (*ZF*), leucine zipper (*LZ*), and forkhead domain (FOX). The PLNLV motif is indicated with a *green bar*. Nuclear localization signals are indicated with *red bars*. A synthetic nine-residue nuclear targeting sequence (*hatched red bars*) was appended to the C-terminus of variants which lack one or both of the endogenous nuclear localization signals. **b** Fluorescence micrographs of HEK293 cells transfected with synthetic truncated FOXP2 variants. Nuclei were stained with Hoechst 33342. **c**, **d** BRET assay for interaction of synthetic truncated FOXP2 variants with full-length FOXP2 and CTBP2. HEK293 cells were transfected with truncated FOXP2 variants fused to *Renilla* luciferase (donor) and FOXP2 (**c**) or CTBP2 (**d**) fused to YFP (acceptor). The control donor protein is a nuclear-targeted luciferase. Values are mean corrected BRET ratios ± S.D. (*n* = 3). *Asterisks* indicate significant differences compared to control (*p* < 0.05, one-way ANOVA followed by Bonferroni post hoc correction). *NS* not significant. Exact *p* values for **c** are 0.002 for FOXP2.330*, 0.025 for FOXP2.259* and <0.0001 for all other variants. Exact *p* values for **d** are 0.001 for FOXP2.423*, 0.012 for FOXP2.330*, 0.97 for FOXP2.259*, and <0.0001 for all other variants

The retention of CTBP binding by the severely truncated p.R328* and p.Q390Vfs*7 variants (Fig. 3d,e) prompted us to investigate further the interaction between FOXP2 and CTBPs. The CTBP binding site in FOXP2 was previously suggested to be a PLNLV motif at residues 422–426, due to the similarity of this motif to the consensus CTBP-binding motif PXDLS (Fig. 4a) [24, 36]. The PLNLV motif is conserved in FOXP1 but not FOXP4, consistent with the lack of interaction between CTBP1 and FOXP4 [24] (Fig. 3a,b). However, the residual interaction of CTBPs with the p.R328* and p.Q390Vfs*7 variants indicates that the PLNLV motif is not essential for the interaction and that the CTBP binding site is at least partially localized to a more N-terminal region of FOXP2 (Fig. 3d,e). Furthermore, one of the yeast two-hybrid screens that identified CTBP1 and CTBP2 as FOXP2 interactors employed a fragment of FOXP2 encompassing residues 122–382, indicating that this region, which lacks the PLNLV motif, is sufficient for the interaction [34].

To map the region of FOXP2 involved in binding to CTBP2, we performed a BRET assay using a series of synthetic truncated versions of FOXP2 (Fig. 4a) [21]. Truncated protein variants lacking the endogenous nuclear localization signals were targeted to the nucleus by addition of an artificial localization signal to the protein C-terminus (Fig. 4a,b). The efficacy of this truncation series in mapping interaction domains was demonstrated using a BRET assay for FOXP2 homodimerization (Fig. 4c). Truncation of FOXP2 after residue 423 did not interfere with homodimerization, whereas truncation after residue 329 dramatically reduced the interaction, reflecting the loss of the critical leucine zipper domain (Fig. 4c) [24].

In a BRET assay for interaction with CTBP2, a FOXP2 variant truncated after residue 487 showed a similar level of interaction to the full-length protein (Fig. 4d), indicating that the C-terminal region of FOXP2 including the FOX domain is not involved in the interaction with CTBPs. More severely truncated forms of FOXP2 showed a progressive reduction in interaction with CTBP2. The substantial retention of interaction between CTBP2 and FOXP2 forms truncated after residue 422 or residue 329 indicates that the PLNLV motif, zinc finger, and leucine zipper domains are not essential for interaction, although they may enhance it, and furthermore, FOXP2 dimerization is not necessary for CTBP2 binding (Fig. 4d). Only the shortest FOXP2 variant, which is truncated after residue 258, showed a complete or near-complete loss of interaction (Fig. 4d). The protein region including residues 259–329 may therefore be sufficient for interaction with CTBP2.

**Fig. 5:**
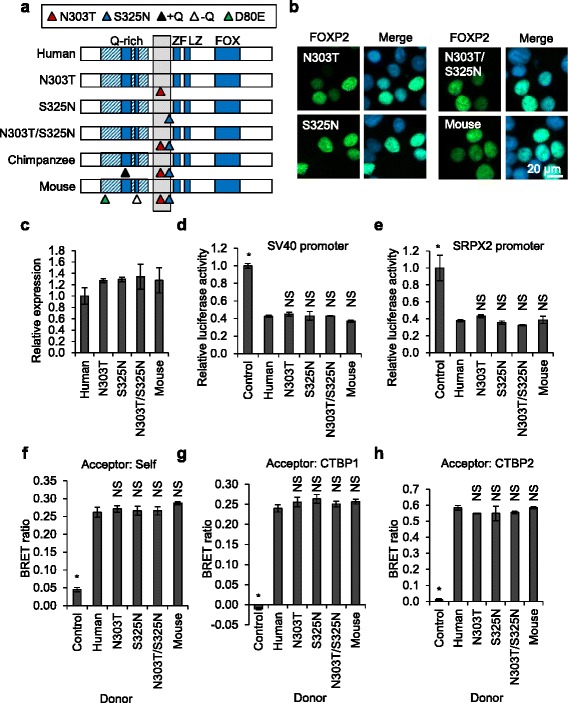
Characterization of FOXP2 variants with ancestral amino acid substitutions. **a** Schematic representation of FOXP2 variants with ancestral amino acid substitutions. The N303T, S325N and N303T/S325N constructs are synthetic variants of human FOXP2 carrying ancestral amino acid substitutions. The chimpanzee and mouse orthologues of FOXP2 are included for comparison. Amino acid differences relative to human FOXP2 are indicated by arrowheads: N303T (*red*), S325N (*blue*), D80E (*green*), glutamine insertion (+Q, *black*), and glutamine deletion (−Q, *white*). Known domains are labelled: glutamine-rich (*Q-rich*) region (*hatched shading*) including polyglutamine tracts (*solid shading*), zinc finger (*ZF*), leucine zipper (*LZ*), and forkhead domain (*FOX*). The minimal CTBP interaction region determined using the BRET assay is indicated by the *grey-shaded box*. **b** Fluorescence micrographs of HEK293 cells transfected with FOXP2 variants with ancestral amino acid substitutions. Nuclei were stained with Hoechst 33342. **c** Fluorescence-based measurement of the expression level of FOXP2 variants with ancestral amino acid substitutions. HEK293 cells were transfected with YFP-FOXP2, together with mCherry for normalization. Fluorescence intensity was measured 48 h post-transfection. Values are mean YFP/mCherry fluorescence ratios ± S.D. (*n* = 3), relative to the value for human FOXP2. **d**, **e** Luciferase reporter assays for transcriptional regulatory activity of FOXP2 variants with ancestral amino acid substitutions. HEK293 cells were transfected with a luciferase reporter vector containing the SV40 promoter (**d**) or the human *SRPX2* promoter (**e**), together with a *Renilla* luciferase normalization plasmid, and YFP-FOXP2 or YFP alone (control). Values are mean relative luciferase activity ± S.D. (*n* = 3), expressed relative to the control. *Asterisks* indicate significant differences compared to human FOXP2 (*p < 0.05, one-way ANOVA followed by Bonferroni post hoc correction). *NS* not significant. Exact *p* values for **d** are <0.0001 for the control, >0.9999 for the N303T, S325N, and N303T/S325N variants and 0.2730 for the mouse protein. Exact *p* values for **e** are <0.0001 for the control and >0.9999 for the N303T, S325N, N303T/S325N and mouse variants. **f**–**h** BRET assays for protein-protein interactions of FOXP2 variants with ancestral amino acid substitutions. HEK293 cells were transfected with FOXP2 variants with ancestral amino acid substitutions fused to *Renilla* luciferase (donor), together with YFP (acceptor) fusions of the same FOXP2 variants (**f**), CTBP1 (**g**), or CTBP2 (**h**). The control donor protein is a nuclear-targeted luciferase and the control acceptor protein is a nuclear-targeted YFP. Values are mean corrected BRET ratios ± S.D (*n* = 3). *Asterisks* indicate significant differences compared to wild-type (WT) FOXP2 (*p* < 0.05, one-way ANOVA followed by Bonferroni post hoc correction). *NS* not significant. Exact *p* values for **f** are <0.0001 for the control, >0.9999 for the N303T, S325N, and N303T/S325N variants and 0.434 for the mouse protein. Exact *p* values for **g** are <0.0001 for the control and >0.9999 for the N303T, S325N, N303T/S325N and mouse variants. Exact *p* values for **h** are <0.0001 for the control, >0.9999 for the N303T, S325N, and mouse variants and 0.10 for the N303T/S325N variant

The region of FOXP2 encompassing residues 259–329 corresponds to a single exon in which non-synonymous nucleotide substitutions between different species are relatively common in comparison to the exons encoding highly conserved elements such as the FOX and leucine zipper domains [39–41]. In particular, this exon includes two non-synonymous nucleotide substitutions that occurred after the split between the human and chimpanzee lineages, but before the split between modern humans and Neanderthals (Fig. 5a) [39, 42]. Mice carrying a ‘humanized’ version of Foxp2, containing these two recent amino acid changes, exhibit altered neuronal morphology and electrophysiology and subtle behavioural changes [43–45]. Strikingly, the morphological and electrophysiological changes have a direction of effect opposite to that observed in mice with only one functional copy of *Foxp2*, suggesting that the changes might have been selected for due to an enhancement of protein function [1, 46].

The molecular mechanisms underlying the neurobiological changes observed in mice carrying humanized *Foxp2* are unknown, but might entail an alteration in the strength of the interaction between Foxp2 and one or more proteins involved in Foxp2-mediated transcriptional regulation. Therefore, to test if the recent amino acid changes affect interaction with CTBPs, we introduced the ancestral amino acid residues, individually and in combination, into human FOXP2 (Fig. 5a). Versions of human FOXP2 carrying the ancestral amino acids were compared with the natural human and mouse proteins in functional assays. Introduction of the ancestral amino acids did not result in any change in protein localization or expression, and furthermore, the human and mouse versions of the protein were indistinguishable in these assays (Fig. 5b,c). Notably, the versions of FOXP2 carrying the ancestral amino acids also did not differ from the natural human protein in assays of transcriptional repression using the SV40 and *SRPX2* promoters, and there was no difference between the human and mouse orthologues in these assays (Fig. 5d,e). Homodimerization was also comparable for all variants tested (Fig. 5f). Finally, the versions of FOXP2 carrying the ancestral amino acids did not differ from the natural human protein in their interaction with CTBP1 and CTBP2 (Fig. 5g,h). Therefore, despite the CTBP binding site in FOXP2 encompassing the protein region that includes the recent amino acid changes, it is unlikely that an alteration in the strength of the interaction between FOXP2 and CTBP1/2 is responsible for the neurobiological differences observed in mice carrying a partially humanized version of Foxp2.

### The FOXP2 polyglutamine tract and neurodevelopmental disorder

**Fig. 6:**
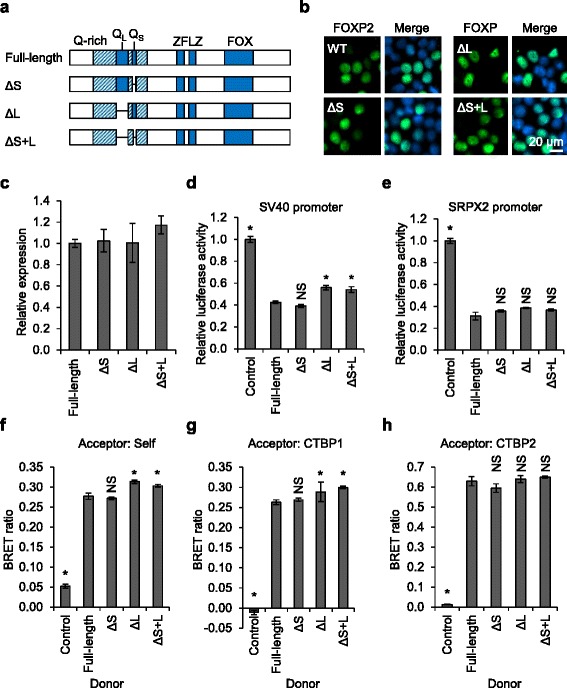
Characterization of synthetic FOXP2 variants with reduced polyglutamine tracts. **a** Schematic representation of synthetic FOXP2 variants with reduced polyglutamine tracts. Known domains are labelled: glutamine-rich (*Q-rich*) region (*hatched shading*) including the long polyglutamine tract (*Q*_*L*_) and short polyglutamine tract (*Q*_*S*_), zinc finger (*ZF*), leucine zipper (*LZ*), and forkhead domain (*FOX*). The Δ*S* variant has a short polyglutamine tract reduced from 10 to 3 residues. The Δ*L* variant has a long polyglutamine tract reduced from 40 to 3 residues. The Δ*S* + *L* variant has shortened versions of both tracts. **b** Fluorescence micrographs of HEK293 cells transfected with FOXP2 variants with reduced polyglutamine tracts. Nuclei were stained with Hoechst 33342. **c** Fluorescence-based measurement of expression level for FOXP2 variants with reduced polyglutamine tracts. HEK293 cells were transfected with YFP-FOXP2 variants together with mCherry for normalization. Fluorescence intensity was measured 48 h post-transfection. Values are mean YFP/mCherry fluorescence ratios ± S.D. (*n* = 3), expressed relative to the value for full-length FOXP2. **d**, **e** Luciferase reporter assays for transcriptional regulatory activity of FOXP2 variants with reduced polyglutamine tracts. Cells were transfected with a luciferase reporter vector containing the SV40 promoter (**d**) or the human *SRPX2* promoter (**e**), together with a *Renilla* luciferase normalization plasmid, and YFP-FOXP2 or YFP alone (control). Values are mean relative luciferase activity ± S.D. (*n* = 3), expressed relative to the control. *Asterisks* indicate significant differences compared to full-length FOXP2 (*p* < 0.05, one-way ANOVA followed by Bonferroni post hoc correction). *NS* not significant. Exact *p* values for **d** are <0.0001 for the control, 0.4892 for Δ*S*, 0.0003 for Δ*L*, and 0.0010 for Δ*S* + *L*. Exact *p* values for **e** are <0.0001 for the control, 0.0768 for Δ*S*, 0.0633 for Δ*L*, and 0.1506 for Δ*S* + *L*. **f**–**h** BRET assays for protein-protein interactions of FOXP2 variants with reduced polyglutamine tracts. HEK293 cells were transfected with FOXP2 variants with reduced polyglutamine tracts fused to *Renilla* luciferase (donor) and YFP (acceptor) fusions of the same variants (**f**), CTBP1 (**g**) or CTBP2 (**h**). The control donor protein is a nuclear-targeted luciferase and the control acceptor protein is a nuclear-targeted YFP. Values are mean corrected BRET ratios ± S.D. (*n* = 3). *Asterisks* indicate significant differences compared to wild-type (WT) FOXP2 (*p* < 0.05, one-way ANOVA followed by Bonferroni post hoc correction). *NS* not significant. Exact *p* values for **f** are <0.0001 for the control, Δ*L* and Δ*S* + *L*, and >0.9999 for Δ*S*. Exact *p* values for **g** are <0.0001 for the control, >0.9999 for Δ*S*, 0.038 for Δ*L*, and 0.024 for Δ*S* + *L*. Exact *p* values for **h** are <0.0001 for the control, 0.4 for Δ*S*, and >0.9999 for Δ*L* and Δ*S* + *L*

FOXP2 contains a large polyglutamine tract of 40 residues (p.Q152-Q191) and a small tract of 10 residues (p.Q200-Q209), separated by 8 residues of intervening polypeptide (Fig. 6a). The tracts are located in the N-terminal portion of the protein, within a larger glutamine-rich region (Fig. 6a). Of note, the large tract in FOXP2 is the longest polyglutamine tract in any human protein in healthy individuals [47]. Abnormal expansions of polyglutamine tracts in at least nine different proteins result in neurodegenerative diseases [48]. However, FOXP2 is not regarded as a strong candidate for involvement in such disorders because its large polyglutamine tract shows relatively little length variation in the human population [47]. The mixture of CAG and CAA codons without long pure CAG repeats probably makes the tract less prone to expansion, and no expansions were detected in a sample of 142 individuals with progressive movement disorders [47, 49]. However, some rare variations in tract length have been observed in individuals with neurodevelopmental disorders (Additional file 4) [8, 11, 13, 14, 17, 18]. Most of the observed variants are deletions of 1–6 residues from the large tract (Additional file 4). Such deletions have also been observed in the general population (Exome Aggregation Consortium (ExAC), Cambridge, MA (http://exac.broadinstitute.org)), suggesting that they are probably not high-penetrance causal variants in cases of severe disorder, but might still represent risk factors, or produce a milder phenotype.

The significance of the polyglutamine tracts to the functions of the FOXP2 protein remains unknown. Polyglutamine tracts may be critical to the function of some proteins [50], and the absence of equivalent tracts in human FOXP1 and FOXP4 (despite presence of glutamine-rich regions) raises the possibility that tract expansion in FOXP2 may have contributed to divergence in FOXP protein function. To investigate the roles of the polyglutamine tracts in FOXP2, we engineered versions of the protein with reduced tract lengths reflecting the presumed ancestral protein sequence. The long tract in FOXP2 underwent expansion early in tetrapod evolution and has subsequently remained relatively stable: ray-finned fish species have a cluster of only 3 glutamine residues in place of the long tract in human FOXP2 [51, 52], whereas the coelacanth, an evolutionary intermediate between ray-finned fish and tetrapods, has a 25-residue tract, and tetrapod species typically have tracts of 35–41 residues (Additional file 5). We therefore reduced the length of the long tract in human FOXP2 to 3 residues. Similarly, the shorter tract was also reduced to 3 residues.

The two tract length reductions were engineered both individually and together (Fig. 6a). The resulting synthetic FOXP2 variants were assayed for a range of molecular properties including subcellular localization (Fig. 6b), expression level (Fig. 6c), transcriptional repression of the SV40 and *SRPX2* promoters (Fig. 6d,e), homodimerization (Fig. 6f), and interaction with CTBPs (Fig. 6g,h). Across these assays, there were no large differences between wild-type FOXP2 and versions of the protein with reduced polyglutamine tracts. Versions of FOXP2 lacking either the large tract or both tracts showed a small but statistically significant reduction in repressive ability in the SV40 luciferase reporter assay and also a small but statistically significant increase in homodimerization and CTBP1 interaction (Fig. 6e,f,g). However, these effects were not consistently observed across independent experiments and therefore may not represent genuine biological differences. The lack of any substantial differences between the wild-type protein and variants with reduced polyglutamine tracts in our assays suggests that the presence of the tracts may not have a marked impact on fundamental aspects of FOXP2 biology.

## Discussion

Our detailed functional characterization of rare FOXP2 variants reported in individuals with neurodevelopmental disorders confirms the etiological role of the Q390Vfs*7 variant, which has similarly deleterious effects on protein function to the two previously characterized pathogenic variants, p.R553H and p.R328*, in our assays. The data presented here point to potential diversity in molecular mechanisms in *FOXP2*-related speech/language disorder. Variants which abolish both transcriptional regulatory activity and protein dimerization, such as the p.R328* and p.Q390Vfs*7 variants, may act as null alleles, resulting in haploinsufficiency of *FOXP2*. In contrast, variants which show a loss of transcriptional regulatory activity but retain the ability to dimerize, like the p.R553H variant, may additionally interfere with the functioning of wild-type protein, as has been suggested for comparable variants in FOXP1 [31]. Variants producing these dominant-negative effects might result in a more severe phenotype. However, it will be necessary to identify further cases of FOXP2-related speech/language disorder resulting from missense variants in order to assess differences in phenotypic outcome.

In our assays, the p.Q17L, p.M406T, p.P416T, or p.N597H variants do not display any substantial effects on the core functions of the FOXP2 protein, suggesting that they may be incidental variants. Notably, the p.N597H variant was previously described as a likely causal variant in a child with CAS [7]. While we cannot rule out that the p.N597H variant disrupts an aspect of FOXP2 function not tested here, subtle effects on protein function are unlikely to lead to a speech/language disorder of comparable severity to complete loss-of-function variants. This case illustrates that, given the high level of rare coding variation in the general population (The 1000 Genomes Project Consortium, 2015), novel rare variants in known disorder-related genes cannot automatically be regarded as causal. This is particularly true if it is not possible to establish segregation of the variant with disorder (or de novo occurrence of a variant in sporadic disorder) or if the disorder is genetically heterogeneous and many genetic risk factors remain unknown, as is the situation for CAS. Functional characterization of novel rare FOXP2 variants is therefore essential to provide a concrete diagnosis of FOXP2-related disorder and to shed light on the aspects of protein function that are disrupted. Our experiments provide a framework for the characterization of novel FOXP2 variants uncovered through future genetic analyses of individuals with speech/language disorder.

Concurrent with the present study, two further cases of probable *FOXP2*-related speech/language disorder were added to the DECIPHER database (patient IDs 271859 and 271246) [53]. One DECIPHER case presented with delayed speech and language development, dysarthria, and pulmonic stenosis and carries a de novo p.R553H variant identical to that observed in the KE family. The probability of recurrence for this particular variant is elevated because it involves mutation of a CpG site. Indeed, pathological mutation events have been observed at the equivalent CpG site in the FOX proteins FOXC2, FOXE1, FOXF1, FOXL2, and FOXP1 [31, 54–57]. The second probable new case of *FOXP2*-related speech/language disorder in DECIPHER presented with delayed speech and language development, delayed fine motor development, strabismus and tall stature and carries a novel de novo stop-gain variant, p.R564*, truncating the FOXP2 protein within the FOX domain. These two new DECIPHER cases underline the characteristic manifestation of heterozygous FOXP2 disruption as a motor speech disorder. In addition, fine motor delays have now been reported in connection with two FOXP2 variants (p.R564* and p.Q390Vfs*7), suggesting that the effects of FOXP2 disruption on motor coordination may in some cases extend beyond the orofacial movements required for speech, consistent with the broader motor skill learning deficits observed in mice with heterozygous FOXP2 disruption [1, 9].

Very recently, while the current manuscript was under review, eight further families with potential *FOXP2*-related speech/language disorder have been newly described, based on sequencing data [58]. Although functional characterizations were not carried out in that study, the new cases suggest that there is some variation in the nature and severity of the speech and language deficits resulting from *FOXP2* disruption and that there may also be variable cognitive impairments and behavioural anomalies in affected individuals. Of particular note, the newly described cohort included two novel missense variants in the FOX domain of FOXP2, but unlike the p.R553H variant found in the KE family, these missense variants do not occur in the DNA-binding helix of the domain. Future functional analyses of these newly identified variants would therefore be valuable to reveal how they might interfere with protein function and to confirm that these variants play a causal role in the disorder. The description of these eight new families represents a step towards a ‘genotype-first’ approach to the characterization of the *FOXP2*-related phenotype [59]. However, sequencing of *FOXP2* in much larger cohorts of language-impaired individuals, preferably coupled to functional analyses, will be necessary to obtain a complete picture of the clinical spectrum associated with *FOXP2*-related disorder and to gain further insights into the role of FOXP2 in neurodevelopment.

In addition to assessing the effects of rare variants on FOXP2 protein function, we performed the first detailed characterization of the interactions between FOXP2 and the transcriptional co-repressors CTBP1 and CTBP2, finding that these interactions are independent of FOXP2 homodimerization and DNA binding. Our results point to an extended CTBP binding site in FOXP2 within residues 258–487, possibly encompassing multiple subsites. Previous yeast two-hybrid screens identified residues 122–382 in FOXP2 as sufficient for interaction with CTBPs [34]. We propose a narrower critical region of 71 residues, from residue 259 to 329. This minimal region contains both of the amino acid substitutions that have occurred since the divergence of the human and chimpanzee lineages. However, we did not find any effect of these substitutions on CTBP interaction, and the human and mouse versions of FOXP2 exhibit comparable levels of interaction with CTBPs. Changes in CTBP interaction do not therefore appear to underlie the neurobiological changes observed in mice carrying a partially humanized version of *Foxp2*. The amino acid changes on the human lineage may instead result in altered interaction with as yet unidentified protein interaction partners or in changes to post-translational modifications. In addition, we confirmed that the FOXP2 paralog FOXP1 also interacts with CTBPs, whereas FOXP4 does not, pointing to a divergence in mechanisms of transcriptional regulation within the FOXP subfamily.

The CTBPs are conserved vertebrate proteins that interact with several different transcription factors and mediate transcriptional repression primarily through recruitment of the histone deacetylases HDAC1/2 [36]. The CTBPs are widely expressed during embryonic development and have essential and partially overlapping roles in the development of multiple organs [35, 36]. They are expressed in the brain from a very early stage, although their specific roles in neurodevelopment have not been extensively investigated [35, 36]. CTBPs may therefore be key players in FOXP2- and FOXP1-mediated transcriptional repression across multiple organs and developmental stages, including in neurodevelopment. In addition, CTBPs may play a role in the post-translational modification of FOXP2. We and other groups have recently demonstrated that FOXP2 is post-translationally modified by SUMOylation [21, 60, 61]. The essential SUMOylation enzyme UBC9 is a core component of the protein complex containing CTBP1, and CTBP1 has been suggested to function as a platform for protein SUMOylation [62]. Furthermore, the SUMO E3 ligase PIAS1, which promotes FOXP2 SUMOylation, is an auxilliary component of the CTBP1 complex [21, 62]. If CTBPs are among the key mediators of FOXP-mediated transcriptional repression, variants in FOXP2 or FOXP1 which disrupt the interaction with CTBPs might have a similar impact on neurodevelopment to variants which disrupt DNA binding, and an assessment of the effects on CTBP binding should therefore be included when characterizing novel, putatively pathogenic variants in FOXP proteins.

Finally, we reported the first detailed analysis of the role of the polyglutamine tracts in FOXP2 function. In our experiments, shortening the polyglutamine tracts in FOXP2 did not have a substantial impact on protein expression, nuclear localization, homodimerization, interaction with CTBPs, or transcriptional repression activity. The tracts do not therefore appear to be essential for core aspects of protein function in cellular assays. However, the tracts may have critical roles in vivo that are not apparent in cell models, and the generation of mice carrying a version of Foxp2 lacking the polyglutamine tracts could therefore be informative. Deletion of the polyglutamine tracts in a small number of other proteins has been found to affect behaviour in mouse models. For example, deletion of the polyglutamine tract in mouse huntingtin results in subtle behavioural changes relating to learning and memory and motor coordination [63]. Replacement of mouse POU3F2 with a *Xenopus* orthologue that lacks polyglutamine tracts and other homopolymer amino acid repeats resulted in neurochemical changes and a dramatic deterioration in pup retrieval behaviour [64]. The polyglutamine tract in mouse SRY has important roles in protein stabilization and transcriptional activation that are essential for sex determination [65]. It is also possible that the polyglutamine tracts in FOXP2 do not have critical roles in normal protein function. The tracts lie within a glutamine-rich protein region that is conserved in FOXP1 and FOXP4 and may be prone to developing expanded stretches of glutamine residues. In FOXP1, a different cluster of glutamine residues has undergone expansion in the rodent lineage to produce tracts of 37 residues in the mouse and 39 in rat, compared to 7 residues in primates. Expansions of up to ~40 glutamine residues within the glutamine-rich region of FOXP proteins may therefore have negligible effect on protein function.

The failure to observe any substantial effects of polyglutamine tract reduction on core aspects of FOXP2 function makes it less likely that tract length reductions contribute to risk for neurodevelopmental disorder. However, one recent study suggested that a FOXP2 polyglutamine tract length variant tentatively associated with speech sound disorder (p.Q172del) exhibits altered transcriptional repression activity in a cell model [18, 66]. The p.Q172del variant was reported to increase transcript levels of the FOXP2 target gene *CNTNAP2* in transfected cells, whereas transcript levels were reduced in cells transfected with wild-type protein [66]. Nonetheless, both the variant and wild-type forms of FOXP2 produced increases in protein levels of CNTNAP2, with the variant having a stronger effect [66]. It is therefore unclear what effect the p.Q172del variant might have on transcriptional regulation in vivo, but our experiments suggest that the effect of a single glutamine deletion is likely to be extremely small. The p.Q172del variant is observed in the general population with a minor allele frequency of 0.6 % in the ExAC database (714 of 115484 chromosomes), and larger tract length reductions are also present in the ExAC database at very low frequency. Studies of larger numbers of individuals with FOXP2 proteins with standard-length and reduced-length tracts are therefore necessary to determine if tract length reduction might confer an increased risk for neurodevelopmental disorder or a reduction in linguistic abilities.

## Conclusions

By performing detailed functional characterization of rare variants in FOXP2 found in individuals with neurodevelopmental disorders, we confirm the causal role of one recently reported uncharacterized variant and provide further characterization of two further causal variants. In addition, we highlight two variants which had been suggested to contribute to the neurodevelopmental disorders in the affected individuals, but which do not have detrimental effects on protein function, suggesting that they are in fact incidental. These findings underline the importance of performing functional characterization of novel variants identified in individuals with neurodevelopmental disorders, even when the affected gene has previously been implicated in disorder. The provision of accurate molecular diagnoses in cases of neurodevelopmental disorder will require consideration of protein function data together with detailed clinical observations and genetic characterization of affected individuals and their parents on a genome-wide scale.

Our research also provides important new insights into the biology of the FOXP2 protein, expanding our knowledge of the molecular functions of this key neural transcription factor. Our characterization of the interaction of FOXP2 with co-repressor proteins of the CTBP family provides additional mechanistic insight into FOXP2-mediated repression of transcription. We also report the first detailed examination of the role of the polyglutamine tracts in FOXP2. We find that these tracts are not essential to the core molecular functions of the FOXP2 protein, suggesting that variations in tract length are unlikely to be a highly penetrant cause of neurodevelopmental disorder.

## Additional files

Additional file 1: DNA sequences of primers used for molecular cloning. (PDF 239 kb)
Additional file 2: DNA sequences of primers used for site-directed mutagenesis. (PDF 234 kb)
Additional file 3: Western blots of YFP-tagged FOXP2 variants. (PDF 521 kb)
Additional file 4: Summary of studies examining FOXP2 polyglutamine tract length variation in individuals with neurodevelopmental and neuropsychiatric disorders. (PDF 251 kb)
Additional file 5: FOXP2 polyglutamine tract lengths in selected vertebrates. (PDF 325 kb)

## Abbreviations

BRET: Bioluminescence resonance energy transfer
CAS: Childhood apraxia of speech
CTBP: C-terminal binding protein
FOX: Forkhead Box
MRI: Magnetic resonance imaging
YFP: Yellow fluorescent protein
