# Supplementary material for: Functional characterization of rare FOXP2 variants in neurodevelopmental disorder
Source: J Neurodev Disord. 2016 Nov 28;8:44. doi: 10.1186/s11689-016-9177-2 (PMC5126810; doi:10.1186/s11689-016-9177-2)
Supplement: Additional file 1: — DNA sequences of primers used for molecular cloning. (PDF 239 kb) [file 11689_2016_9177_MOESM1_ESM.pdf]

Additional file 1 DNA sequences of primers used for molecular cloning

Construct	Forward primer[a]	Site	Reverse primer[a]	Site
CTBP2	GAGGATCCTGGCCCTTGTG GATAAGCACAAAGTC	BamHI	AGTCTAGACTATTGCTCGTTGGG GTGCTCTCGATTG	XbaI
FOXP2 R328*	AGGGATCCAAGAATCTGGG ACTGAGACA	BamHI	CTCTAGATTATCTTGCACTTAGA ACTGAAG	XbaI
FOXP2 670*	AGGGATCCAAGAATCTGGG ACTGAGACA	BamHI	GTCTAGATTATATGTGCGGCTGA GGTGAGC	XbaI
FOXP2 614*	AGGGATCCAAGAATCTGGG ACTGAGACA	BamHI	GTCTAGATTACTGCAAACTGGCA TTAAGAG	XbaI
FOXP2 488*	AGGGATCCAAGAATCTGGG ACTGAGACA	BamHI	GTCTAGACATGGGAATGTTGTAT TTGTC	XbaI
FOXP2 423*	AGGGATCCAAGAATCTGGG ACTGAGACA	BamHI	GTCTAGAAGGTTTGGGAGATGG TTTGGG	XbaI
FOXP2 330*	AGGGATCCAAGAATCTGGG ACTGAGACA	BamHI	GTCTAGAGTCTCGTCTTGCACTT AGAAC	XbaI
FOXP2 259*	AGGGATCCAAGAATCTGGG ACTGAGACA	BamHI	GTCTAGATTGAGGCAGCGATTG GACAGG	XbaI

[a] Restriction sites are underlined
