# Supplementary material for: Functional characterization of rare FOXP2 variants in neurodevelopmental disorder
Source: J Neurodev Disord. 2016 Nov 28;8:44. doi: 10.1186/s11689-016-9177-2 (PMC5126810; doi:10.1186/s11689-016-9177-2)
Supplement: Additional file 2: — DNA sequences of primers used for site-directed mutagenesis. (PDF 234 kb) [file 11689_2016_9177_MOESM2_ESM.pdf]

Additional file 2 DNA sequences of primers used for site-directed mutagenesis

Variant	Primer 1	Primer 2
p.Q17L	GCAACAGTTCAATGAATCTAAATGGAATGAGCACTCTAAG	GCTTAGAGTGCTCATTCCATTTAGATTCATTGAACTGTTGC
p.Q390Vfs*7	TGCGACCCTCAGAGACCAAACCATCTCCC	TAGAAAGCTGTATTTCTAACTTGCACCACCTGCATTTGC
p.M406T	ACGTCTTCAAGCAATGACGACCCACTTGCACATG	GCATGTGCAAGTGGGTCGTCATTGCTTGAAGACGT
p.P416T	TGCGACCCTCAGAGACCAAACCATCTCCC	GGGAGATGGTTTGGTCTCTGAGGGTCGCA
p.R553H	CTTGGAAGAATGCAGTACATCATAATCTTAGCCTGCAC	GTGCAGGCTAAGATTATGATGTACTGCATTCTTCCAAG
p.N597H	ATAACAGGAAGTCCAACCTTAGTAAAACATATACCAGTTTTAG	CTAAACTGGTAGGTATATGTTTTACTAAGGTTGGACTTCCTGTTAT
p.N303T	CTCGACTACCTCCTCCACCACTTCCAAAGCATCAC	GTGATGCTTTGGAAGTGGTGGAGGAGGTAGTCGAG
p.S325N	GTGAATGGACAGTCTTCAGTTCTAAATGCAAGACGAGAC	GTCTCGTCTTGCATTTAGAACTGAAGACTGTCCATTCAC
