# Supplementary material for: Functional characterization of rare FOXP2 variants in neurodevelopmental disorder
Source: J Neurodev Disord. 2016 Nov 28;8:44. doi: 10.1186/s11689-016-9177-2 (PMC5126810; doi:10.1186/s11689-016-9177-2)

Additional file 3 Western blots of YFP-tagged FOXP2 variants

HEK293 cells were transfected with YFP-FOXP2 and blots of whole cell lysate were probed with anti-YFP to detect FOXP2 and with anti-β-actin to confirm equal loading.

Rare FOXP2 variants found in individuals with neurodevelopmental disorders.

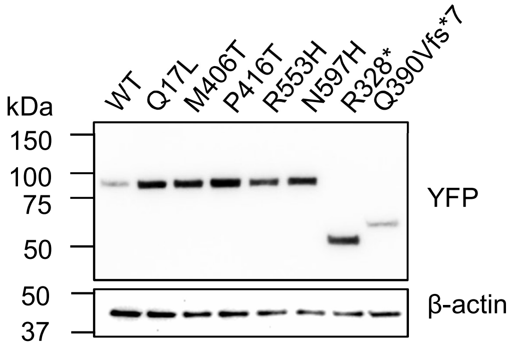

Synthetic truncated forms of FOXP2

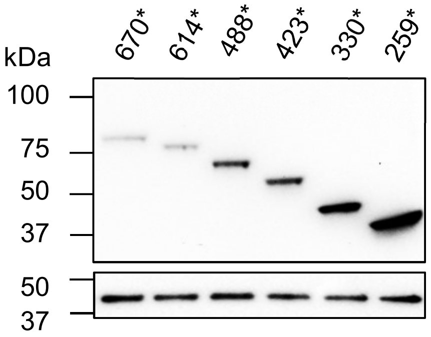

FOXP2 variants with ancestral amino acid substitutions

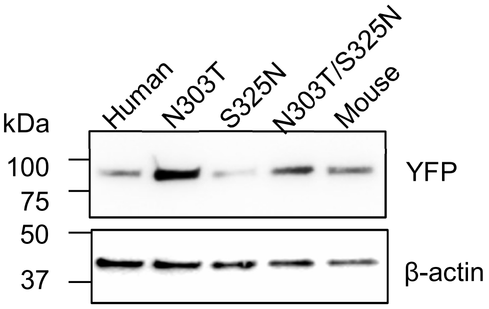

Synthetic FOXP2 variants with reduced polyglutamine tracts

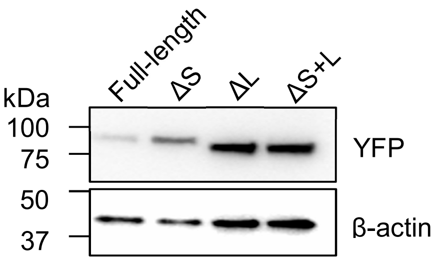

Supplement: Additional file 3: — Western blots of YFP-tagged FOXP2 variants. (PDF 521 kb) [file 11689_2016_9177_MOESM3_ESM.pdf]
