# Supplementary material for: Functional characterization of rare FOXP2 variants in neurodevelopmental disorder
Source: J Neurodev Disord. 2016 Nov 28;8:44. doi: 10.1186/s11689-016-9177-2 (PMC5126810; doi:10.1186/s11689-016-9177-2)
Supplement: Additional file 4: — Summary of studies examining FOXP2 polyglutamine tract length variation in individuals with neurodevelopmental and neuropsychiatric disorders. (PDF 251 kb) [file 11689_2016_9177_MOESM4_ESM.pdf]

**Additional file 4** Summary of studies examining FOXP2 polyglutamine tract length variation in individuals with neurodevelopmental and neuropsychiatric disorders

| **Individuals screened** | **Polyglutamine tract variations observed** | **Reference** |
|---|---|---|
| 48 probands from multiplex autism families, 43 probands from SLI families. | 10Q->12Q in one proband with SLI; inherited but did not segregate with disorder. | Newbury et al. (2002) |
| 75 families with sibling pairs with autism + 60 independent autistic probands. 160 controls. | 40Q->34Q in mother and proband of one trio. 40Q->35Q in mother and both probands of one affected sibling pair family. | Wassink et al. (2002) |
| 72 autism cases + 98 controls. | No variation observed. | Gauthier et al. (2003) |
| 53 autism cases + 50 controls. | 40Q->39Q in 4 cases and 2 controls. | Li et al. (2005) |
| 49 CAS probands. | 40Q->44Q in one proband; absent from affected sibling. Parental genotypes unknown. | MacDermot et al. (2005) |
| 247 schizophrenia cases, 98 autism cases, 56 idiopathic intellectual disability cases, 314 controls. | 40Q->36Q in one autism case; inherited from father.<br>40Q->44Q in one autism case. Parental genotypes unknown. | Laroche et al. (2008) |
| 150 speech sound disorder cases + 120 controls. | 40Q->39Q in 5 cases. | Zhao et al. (2010) |
| 293 schizophrenia cases + 340 controls. | No variation observed. | Tolosa et al. (2010) |
| 95 schizophrenia cases. | No variation observed. | Levchenko et al. (2014) |

SLI, specific language impairment
CAS, childhood apraxia of speech.
