# Supplementary material for: Functional characterization of rare FOXP2 variants in neurodevelopmental disorder
Source: J Neurodev Disord. 2016 Nov 28;8:44. doi: 10.1186/s11689-016-9177-2 (PMC5126810; doi:10.1186/s11689-016-9177-2)
Supplement: Additional file 5: — FOXP2 polyglutamine tract lengths in selected vertebrates. (PDF 325 kb) [file 11689_2016_9177_MOESM5_ESM.pdf]

Additional file 5 FOXP2 polyglutamine tract lengths in selected vertebrates

Taxon	Species	Number of Q residues[a]		UniProt
		Long tract	Short tract	
Primates	Human *(Homo sapiens)*	40	10	O15409
	Chimpanzee *(Pan troglodytes)*	41	10	Q8MJA0
	Bonobo *(Pan paniscus)*	41	10	Q8HZ00
	Gorilla *(Gorilla gorilla gorilla)*	38	10	Q8MJ99
	Baboon *(Papio anubis)*	39	10	A9RAA3
	Orangutan *(Pongo pygmaeus)*	39	9	Q8MJ98
	Orangutan *(Pongo abelii)*	39	9	H2PN93
	Gibbon *(Hylobates lar)*	39	9	Q5QL03
	Gibbon *(Nomascus leucogenys)*	39	10	G1RL29
	Macaque *(Macaca mulatta)*	39	10	Q8MJ97
	Marmoset *(Callithrix jacchus)*	38	10	F7IHC2
	Bushbaby *(Otolemur garnettii)*	38	10	B5FWC6
Non-primate mammals	Mouse *(Mus musculus)*	40	9	P58463
	Rat *(Rattus norvegicus)*	36	9	P0CF24
	Opossum *(Monodelphis domestica)*	39	5	F7F0A1
	Platypus *(Ornithorhynchus anatinus)*	36	9	F7F7P1
Birds and reptiles	Chicken *(Gallus gallus)*	38	5	F1NY57
	Zebra finch *(Taeniopygia guttata)*	38	8	K4EPC4
Amphibians	Frog *(Xenopus laevis)*	35	3	Q4VYS1
	Frog *(Xenopus tropicalis)*	38	2	A4IIF5
	Turtle *(Pelodiscus sinensis)*	37	3	K7G4L3
Lobe-finned fish	Coelacanth *(Latimeria chalumnae)*	25	6	H3AY31[b]
Ray-finned fish	Zebrafish *(Danio rerio)*	3	7	Q4JNX5
	Pufferfish (*Takifugu rubripes)*	3	6	V6F872
	Pufferfish *(Tetraodon nigroviridis)*	3	6	V6F979
	Tilapia *(Oreochromis niloticus)*	3	7	V6F808

[a] Determined using protein sequences from the UniProt database
[b] This protein sequence is not annotated as FOXP2 in the UniProt database but is identified as a 1-to-1 orthologue of human FOXP2 by the Ensembl genome browser.
